# Current status and influencing factors of medication behavior among children and adolescents: a cross-sectional study in China

**DOI:** 10.3389/fpubh.2025.1583645

**Published:** 2025-07-30

**Authors:** Ximu Sun, Lijie Yang, Qin Han, Yixin Sun, Han Zhou, Xiaolin Xu, Peng Guo

**Affiliations:** ^1^Department of Pharmacy, National Center for Children’s Health, Beijing Children's Hospital, Capital Medical University, Beijing, China; ^2^Department of Pharmacy, The Sixth Affiliated Hospital of Harbin Medical University, Harbin, China; ^3^College of Health Management, Shandong University of Traditional Chinese Medicine, Jinan, China; ^4^Clinical Research Center, National Center for Children’s Health, Beijing Children's Hospital, Capital Medical University, Beijing, China; ^5^Key Laboratory of Major Diseases in Children, Ministry of Education, Beijing Children’s Hospital, Capital Medical University, National Center for Children’s Health, Beijing, China

**Keywords:** adolescents, children, health management, medication behavior, medication literacy, temporal self-regulation theory

## Abstract

**Background:**

Children face higher medication risks than adults, making safe and rational drug use a societal concern. For children aged 8 and older, early intervention is key to fostering responsible medication habits. This study, grounded in Temporal Self-Regulation Theory (TST), suggests that temporal valuations, behavioral prepotency, and self-regulatory capacity influence medication behaviors, with weaker self-regulation potentially linked to stronger behavioral prepotency.

**Aims:**

This study examined medication behavior patterns among Chinese children and adolescents, identified the factors influencing these behaviors, and evaluated the applicability of the TST in elucidating medication behavior.

**Methods:**

This study surveyed 4,288 children and adolescents aged 8–18 from October 2023 to May 2024, recruited from children’s hospitals in 18 cities across mainland China. Data on demographics, health literacy, medication self-efficacy, perceived stress, and medication behavior were collected via questionnaires. Single-factor and multiple linear regression analyses identified significant factors and their contributions to the outcome.

**Results:**

A total of 4,044 questionnaires were collected. The sample included 53.07% males and 46.93% females, with a mean age of 13.60 ± 2.85 years. The mean scores of medication behavior, medication literacy, perceived stress, and medication self-efficacy were 109.49 ± 17.57, 36.00 ± 9.86, 6.55 ± 2.82, 9.24 ± 4.12, respectively. Medication behavior correlated positively with medication literacy (*r* = 0.420, *p* < 0.001) and negatively with perceived stress (*r* = −0.299, *p* < 0.001) and medication self-efficacy (*r* = −0.403, *p* < 0.001). Self-efficacy was measured using reverse-scored items, with lower scores corresponded to higher levels of self-efficacy. Multiple linear regression analysis identified age (*β* = 0.093, *p* < 0.001), gender (*β* = −0.058, *p* < 0.001), residence within the past 3 months (*β* = 0.043, *p* = 0.004), having a separate room (*β* = 0.027, *p* = 0.039), medication literacy (*β* = 0.259, *p* < 0.001), perceived stress (*β* = −0.214, *p* < 0.001), and medication self-efficacy (*β* = −0.238, *p* < 0.001) as significant predictors of medication behavior.

**Conclusion:**

The study emphasizes that perceived stress, medication literacy, and self-efficacy significantly influence medication behavior in children and adolescents. Targeted interventions in stress management, literacy improvement, and self-efficacy enhancement could foster safer medication use, warranting further research for development and testing.

## Introduction

With the dissemination of medical knowledge, the practice of self-medication within households has become increasingly prevalent in China. Pediatric patients represent a unique and vulnerable population in pharmacotherapy, primarily due to their immature and evolving organ and enzyme systems (1). The physiological distinctiveness of pediatric populations leads to pharmacokinetic profiles that differ significantly from those observed in adults. This difference is correlated with an increased risk of adverse reactions or harm. Furthermore, there is a notable scarcity of clinical trials and a lack of diversity in the formulation, dosage forms, and specifications of pediatric medications, leading to medication error rates ranging from 30 to 80% (2). Ensuring the safety and efficacy of medication for pediatric populations constitutes a critical priority in healthcare, requiring clinicians to implement appropriate therapeutic strategies to safeguard the well-being of children. Children above the age of eight exhibit heightened self-awareness and are progressively moving towards self-management. Early intervention during this transition to independent decision-making is crucial for cultivating rational medication behaviors. This developmental stage is pivotal for acquiring the self-regulatory skills necessary to ensure safe and effective medication practices in adulthood. There exists a process linking the generation of motivation to the implementation of appropriate behavior, with time playing a crucial role in this progression. To offer a novel temporal perspective for analyzing group behavioral differences and to uncover dynamic mechanisms often overlooked in traditional research, we have innovatively incorporated theoretical models in our exploration of influencing factors.

Temporal Self-Regulation Theory (TST) represents a novel theoretical framework to elucidate individual health behaviors. This theory synthesizes the temporal aspects of behavioral contingencies with self-regulatory capacities to address the enduring gap between intention and behavior (3). The theory posits that behaviors aligned with long-term interests arise from the complex interplay of biological predispositions, cognitive assessments, and social-contextual influences. In particular, TST proposes that behavioral engagement is determined by the combined influence of perceived outcome value and temporal discounting. The theory has been applied to elucidate a range of various health behaviors in empirical research, encompassing heavy episodic drinking (4), daily snacking (5), both healthy and unhealthy eating habits (6), as well as medication adherence (7). In contrast to the Theory of Planned Behavior, which assumes that behaviors are directly influenced by intention (8), TST recognizes that behavior encompasses a self-regulatory process that cannot be entirely explained by planning alone. Additionally, although the Health Belief Model emphasizes perceived threats and benefits related to health behaviors (9), it does not adequately address the behavioral dimensions, particularly the dynamic and temporal aspects of behavior regulation. In contrast, TST provides a more comprehensive framework that effectively elucidates the intention-behavior gap and highlights the role of self-regulation in resisting immediate temptations to achieve long-term health benefits. This makes TST particularly suitable for examining medication behavior in the management of chronic diseases.

TST assumes that the temporal dispersion of costs and benefits associated with health behaviors is perceived at the individual level as a cognitive phenomenon known as *temporal contingency*. For instance, the prevention of chronic diseases requires long-term interventions, the effects of which may not become apparent until years or even decades in the future (3). Individuals can regulate their behavior appropriately only by acknowledging the connection between present actions and future outcomes. In this study, we employed the concept of medication literacy to represent execution intention from a temporal perspective. Medication literacy, a subset of health literacy specific to the pharmacy domain, was first introduced in a UK government document in 2005 (10). It refers to an individual’s capacity to acquire, comprehend, communicate, calculate, and process patient-specific medication information, enabling them to make informed decisions regarding their medications and overall health, thereby ensuring the safe and effective use of their prescribed treatments. It is a fundamental determinant in assessing the medication-related behaviors of individuals with chronic diseases, demonstrating a positive correlation with such behaviors (11, 12). Studies indicate that individuals with lower medication literacy are 1.5 to 3 times more likely to encounter adverse health outcomes compared to those possessing higher levels of medication literacy (13). Nonetheless, the interplay between medication literacy and complex social determinants, and its impact on the medication behaviors of children and adolescents, remains insufficiently comprehended.

Within the framework of the TST, *behavioral prepotency* denotes the tendency of individuals to prioritize certain behaviors in specific contexts due to habitual or automatic responses. Empirical evidence suggests that past behavior is one of the most robust predictors of future behavior, sometimes surpassing the predictive efficacy of behavioral intentions and other well-established social-cognitive constructs (3). To quantify this phenomenon, we employed a validated, self-constructed medication self-efficacy scale. *Self-regulatory capacity*, defined as the ability to establish goals, monitor progress, and adjust strategies to overcome internal or external disruptions, was assessed in this study using the Perceived Stress Scale (PSS).

Although the Theory of TST has demonstrated substantial utility and explanatory power in health behavior research, previous studies have not systematically applied its fundamental constructs—*temporal contingency, behavioral prepotency, and self-regulatory capacity* —to the context of pediatric medication behavior. This research seeks to demonstrate the theory’s prescriptive utility in guiding the selection of variables and to enhance the theory’s generalizability and applicability in this specific domain. Consequently, the current study seeks to investigate the prevailing state of medication behavior among children and adolescents in China and to assess the applicability of the TST in understanding medication behavior within the frameworks of medication literacy and medication self-efficacy. Based on the theoretical assumptions of TST, the following hypotheses were formulated: H1: Temporal contingency, behavioral prepotency, and self-regulatory capacity will account for additional variance in medication behavior. H2: The weaker self-regulatory capacity would be associated with stronger behavioral prepotency.

## Materials and methods

### Study design

This study is a large-scale, multicenter cross-sectional survey executed in Mainland China from October 2023 to May 2024, titled Medication Literacy Investigation of Chinese Children (MLICC). The study encompasses pediatric hospitals across 23 provinces, 5 autonomous regions, and 4 municipalities. Stratified random sampling was used at the provincial, community, or village level, whereas nonproportional quota sampling was utilized at the community, village, or individual level. Ethical approval for this research was granted by the Ethics Committee of Beijing Children’s Hospital, affiliated with Capital Medical University (Ethics Approval Number: 2023-E-015-R).

To ensure the representativeness and reliability of the sample population, a combination of probability sampling methods for hospitals and quota sampling methods for individuals was utilized. The minimum sample size for each layer was achieved through the SPSS random sampling method: *n* = μ*_α_* 2*π* (1-π)/*δ*^2^, δ = p-π (α = 0.05, μ_α_ = 1.96, δ = 0.05, π = 0.50). Due to the unknown total population, π took a value of 50%, μ_α_ = 1.96, δ = 0.10, giving *n* = 345.7 (approximately 346 samples). To reduce the impact of invalid questionnaires on the results, the missing rate was set at 10%, which means an additional 10% of respondents are required, giving *n* = 346*1.1 = 380.6 (approximately 381 samples). Considering the sample’s persuasiveness, the questionnaire’s response rate, and the attrition rate, this study determined that a minimum of 50 samples per hospital was adequate, making the sample size sufficient.

### Participants

The researchers evaluated 4,288 individuals who satisfied the inclusion criteria and subsequently distributed electronic questionnaires. A total of 4,044 valid questionnaires were collected, yielding a response rate of 94.3%, which encompasses 320 pre-test responses. The eligibility criteria for the study were as follows: (1) participants were required to be between the ages of 8 and18 years; (2) participants voluntarily participated in the study, with informed consent obtained from either the participants themselves or their legal guardians; (3) participants had to be nationals of the People’s Republic of China; (4) they were required to be permanent residents of China, annual foreign travel not exceeding 1 month; (5) participants needed the capability to independently complete the questionnaire or do so with assistance from their guardians; and (6) they had to possess an understanding of the questionnaire items. The exclusion criteria included: individuals with mental abnormalities or cognitive impairments, those currently engaged in other related studies, and those unwilling to participate.

### Data collection

The study established dedicated survey sites at each participating children’s hospital. The investigators recruited children aged 8–18 years who met the eligibility criteria as participants and administered electronic questionnaires in person. The questionnaires were accessible via QR code scanning. Participants completed the questionnaire by clicking the provided link, and informed consent was obtained prior to the survey. For participants with cognitive ability but insufficient ability to complete the questionnaire independently, the investigators conducted one-on-one interviews and recorded responses on their behalf. In cases where younger children were present and might not fully comprehend the questionnaire, investigators were available to provide in-person explanations of the content.

### Measures

#### TST variables

*Demographic data* were gathered via electronic questionnaires that included variables such as gender, body mass index (BMI), educational attainment, history of chronic diseases, residence within the past 3 months, registered permanent residence, status as an only child, presence of cohabitants on long-term medication regimens, cohabitants’ employment as healthcare professionals, and the availability of a separate room within the household. Chronic diseases refer to the general term for diseases that are not contagious and persist for a prolonged period - typically 3 months or more (e.g., heart diseases, diabetes, chronic respiratory diseases, and cancers).

*Temporal contingency* was operationalized through the assessment of medication literacy using the 12-item Short-Form Health Literacy scale (HLS-SF12). The HLS-SF12 instrument encompasses three dimensions: health care, disease prevention, and health promotion, comprising 12 items, each evaluated on a 4-point Likert scale (1 = very difficult, 2 = difficult, 3 = easy, 4 = very easy). A standardized health literacy (HL) index, ranging from 0 to 5, is computed using the formula: index = (mean-1) * (50/3), where higher index values indicate greater levels of medication literacy. In this study, the Cronbach’s *α* was 0.933 (14).

*Behavioral prepotency* was assessed utilizing medication self-efficacy scales, which encompassed two dimensions of self-efficacy and intensity, evaluated on a 5-point Likert scale (ranging from 1, indicating strong agreement, to 5, indicating strong disagreement). The scale comprised four items, yielding a cumulative score ranging from 5 to 20 points. Self-efficacy was assessed through the use of reverse-scored items, whereby lower scores corresponded to higher levels of self-efficacy. This scale, developed by the research team, demonstrated robust reliability and validity with a Cronbach’s *α* of 0.766. Detailed contents of the scale are provided in the Supplementary material.

*Self-regulatory capacity* was assessed using the 4-item Perceived Stress Scale (PSS-4), which serves as a comprehensive instrument for evaluating stress [Cronbach’s α = 0.82 (15)]. This scale encompasses two dimensions, specifically measuring unpredictability and uncontrollability and employs a 5-point Likert scale ranging from 0 to 4, where 0 indicates “never” and 4 indicates “always.” Elevated scores on the PSS were found to be positively correlated with increased levels of perceived stress.

*Behavior* was evaluated in terms of medication-related practices across six domains: label comprehension (e.g., “I pay attention to the dosage and administration in the medicine instructions”), recognition of adverse reactions [e.g., “If gastrointestinal symptoms such as nausea or diarrhea occur after taking medication, I consider a potential link to the administered drug”], medication adherence (e.g., “I often forget to take my medications”), administration methods (e.g., “I consumed medications with liquids other than water, such as tea or cola”), unsafe medication use (e.g., “I take antipyretic analgesics, such as ibuprofen, more than four times a day”), and difficulties in medication intake (e.g., “I feel anxious before taking medication”). The instrument comprised a total of 26 items, each rated on a 5-point Likert scale ranging from 1 (strongly disagree) to 5 (strongly agree). Notably, three subscales—medication adherence, unsafe medication, and difficulty taking medication—were reverse-scored. The overall score was calculated by summing the scores of all items across the various dimensions. Higher scores were indicative of safe medication behavior among the participants. The overall Cronbach’s *α* coefficient for the scale is 0.914, with individual dimension coefficients ranging from 0.792 to 0.941 (16). Details of the scale are provided in the Supplementary material.

### Statistical analysis

Data analysis was performed with SPSS version 19.0 and R 4.4.2. Quantitative data were presented as the mean ± standard deviation (SD). For qualitative data, the number and percentage (%) of cases were reported. Independent samples *t*-tests were employed to examine group differences in the mean scores of medication behavior for binary variables. For polytomous variables, a one-way analysis of variance (ANOVA) was conducted to assess overall between-group differences in the mean scores of medication behavior. A *p*-value of less than 0.05 was considered to indicate statistical significance. To provide a measure of the practical significance of the observed differences, effect sizes were computed. Cohen’s d was applied to quantify the standardized mean difference of two groups, while eta squared (η^2^) was used to evaluate the effect size in the context of multi-categorical variables. The correlations between variables were assessed using Spearman’s rank correlation coefficients. The significant factors (*p* < 0.1) were further evaluated using multiple linear regression analysis. The regression coefficients (B), along with their 95% confidence intervals (95% CI) and standard errors (SE) were estimated to quantify effect sizes and precision. Standardized regression coefficients (Beta) were calculated to compare the relative importance of predictors on a common scale. The statistical significance of each predictor was evaluated using *t*-tests, with a threshold of *p* < 0.05 indicating statistical significance. Variance inflation factors (VIF) were computed to assess multicollinearity among the independent variables, with a VIF greater than 5 suggesting potential collinearity.

## Results

### The demographic characteristics and univariate analyses

A total of 4,044 participants were included with an average age of 13.6 years old. The score of medication behavior was 109.49 ± 17.57. The participants were mostly male (53.07%), with a junior high school or elementary school education (32.02%, respectively), without chronic diseases (90.96%), living in the city in the last 3 months (68.32%), registered permanent residence located in the countryside (51.23%), only child (68.28%), cohabitants without long-term medication (78.50%), cohabitants were not healthcare professionals (83.92%), and the absence of a separate room (85.42%). The results of the univariate analyses are presented in Table 1. Independent samples *t*-test and ANOVA showed that having chronic diseases (*p* = 0.013), living in the countryside in the last 3 months (*p* < 0.001), registered permanent residence in the countryside (*p* = 0.034), and the absence of a separate room (*p* < 0.001) were significantly associated with lower medication behavior scores. Only the variable ‘had a separate room’ had a small effect size, the other variables had a less than small effect size.

**Table 1:** Demographic characteristics and univariate analyses of factors associated with medication behavior scores in Chinese children and adolescents (*N* = 4,044).

Characteristics	*N* (%)	Mean (SD)	t/F	*p*	*d*
Gender			−0.297	0.766	0.078
Male	2,337 (53.07)	110.22 ± 17.29			
Female	2067 (46.93)	108.85 ± 17.79			
Educational attainment			1.268^a^	0.057	0.018^b^
Primary school	1,410 (32.02)	106.85 ± 17.89			
Junior high school	1,410 (32.02)	112.22 ± 17.35			
Secondary school	612 (13.89)	107.32 ± 18.20			
High school	852 (19.35)	110.83 ± 16.38			
Junior college	76 (1.73)	110.14 ± 14.92			
Tertiary school	44 (0.99)	109.91 ± 16.82			
Chronic diseases			2.473	0.013*	−0.130
Yes	398 (9.04)	107.42 ± 17.16			
No	4,006 (90.96)	109.70 ± 17.60			
Residence within the past 3 months			−4.127	< 0.001**	−0.134
Countryside	1,395 (31.68)	107.89 ± 17.59			
City	3,009 (68.32)	110.24 ± 17.52			
Registered permanent residence			−2.126	0.034*	−0.064
Countryside	2,256 (51.23)	108.94 ± 17.44			
City	2,148 (48.77)	110.07 ± 17.69			
Only child			−1.232	0.218	0.040
Yes	3,007 (68.28)	109.72 ± 17.38			
No	1,397 (31.72)	109.01 ± 18.00			
Cohabitants on long-term medications			−0.117	0.907	0.004
Yes	947 (21.50)	109.55 ± 16.95			
No	3,457 (78.50)	109.48 ± 17.74			
Cohabitants’ employment as healthcare professionals			1.692	0.091	−0.069
Yes	708 (16.08)	108.47 ± 17.86			
No	3,696 (83.92)	109.69 ± 17.51			
Had a separate room			−6.157	< 0.001**	0.258
Yes	642 (14.58)	110.16 ± 17.45			
No	3,762 (85.42)	105.56 ± 17.86			

Mean (SD): results of medication behavior scores; d: results from Cohen’ d. *p < 0.05, **p < 0.01.

a ANOVA, analysis of variance.

b Eta squared.

### Means, standard deviations and correlations between variables

The mean scores of medication literacy, medication self-efficacy, and PSS-4 were 36.00 ± 9.86, 9.24 ± 4.12, and 6.55 ± 2.82, respectively (Table 2). The total mean score of medication behavior was 109.49 ± 17.57, with 6-dimension mean scores of 23.98 ± 5.75 for label comprehension, 14.56 ± 4.24 for recognition of adverse reactions, 22.90 ± 5.79 for medication adherence, 11.08 ± 3.19 for administration methods, 29.52 ± 6.75 for unsafe medication, and 7.46 ± 2.32 for difficulties in medication intake. The correlation coefficient matrix of the variables is displayed in Table 2. Medication behavior was significantly positively correlated with age (*r* = 0.088, *p* < 0.001) and medication literacy (*r* = 0.420, *p* < 0.001) and negatively correlated with perceived stress (*r* = −0.299, *p* < 0.001) and medication self-efficacy (*r* = −0.403, *p* < 0.001). BMI was not significantly positively correlated with medication behavior (*r* = 0.017, *p* = 0.271). Medication literacy was positively correlated with age (*r* = 0.092, *p* < 0.001) and BMI (*r* = 0.039, *p* = 0.010), and negatively correlated with perceived stress (*r* = −0.208, *p* < 0.001) and medication self-efficacy (*r* = −0.443, *p* < 0.001). Medication self-efficacy was negatively correlated with age (*r* = −0.066, *p* < 0.001) and BMI (*r* = −0.051, *p* = 0.001), whereas it was positively correlated with perceived stress (*r* = 0.211, *p* < 0.001). Perceived stress was positively correlated with age (*r* = 0.200, *p* < 0.001) and BMI (*r* = 0.080, *p* < 0.001).

**Table 2:** Means, standard deviations and correlations between variables.

Variables	*M*	SD	Age	BMI	Perceived stress	Medication self-efficacy	Medication literacy	Medication behavior
Age (years)	13.60	2.85	1.000					
BMI (kg/m^2^)	19.66	3.91	0.248^**^	1.000				
Perceived stress (scores)	6.55	2.82	0.200^**^	0.080^**^	1.000			
Medication self-efficacy (scores)	9.24	4.12	−0.066^**^	−0.051^**^	0.211^**^	1.000		
Medication literacy (scores)	36.00	9.86	0.092^**^	0.039^*^	−0.208^**^	−0.443^**^	1.000	
Medication behavior (scores)	109.49	17.57	0.088^**^	0.017	−0.299^**^	−0.403^**^	0.420^**^	1.000

**p < 0.01, *p < 0.05; BMI, body mass index.

### Multiple linear regression analysis

The assignment of the independent variables is displayed in Table 3. Significant factors in the final results, as shown in Table 4, include age (*β* = 0.093, *p* < 0.001), gender (*β* = −0.058, *p* < 0.001), residence in the past 3 months (*β* = 0.043, *p* = 0.004), had a separate room (*β* = 0.027, *p* = 0.039), medication literacy (*β* = 0.259, *p* < 0.001), perceived stress (*β* = −0.214, *p* < 0.001), medication self-efficacy (*β* = −0.238, *p* < 0.001).

**Table 3:** Assignment of independent variables.

Variables	Assignment method
Age	Original value input
Gender	Male = 1, Female = 0
Chronic diseases	Yes = 1, No = 0
Residence within the past 3 months	City = 1, Countryside = 0
Registered permanent residence	City = 1, Countryside = 0
Had a separate room	Yes = 1, No = 0
Medication literacy	Original value input
Perceived stress	Original value input
Medication self-efficacy	Original value input

**Table 4:** Multiple linear regression analysis.

Variables	B [95% CI]	SE	Beta	*t*	*p*	VIF
(Constant)	102.295 [98.658, 105.932]	1.855		55.147	< 0.001**	
Age	0.571 [0.407, 0.735]	0.084	0.093	6.812	< 0.001**	1.006
Gender	−2.026 [−2.911, −1.142]	0.451	−0.058	−4.492	< 0.001**	1.134
Chronic diseases	1.263 [−0.291, 2.817]	0.793	0.021	1.593	0.111	1.025
Residence within the past 3 months	1.623 [0.506, 2.741]	0.570	0.043	2.848	0.004**	1.396
Registered permanent residence	−0.623 [−1.662, 0.415]	0.530	0.018	−1.177	0.239	1.392
Had a separate room	1.329 [0.064, 2.594]	0.645	0.027	2.060	0.039*	1.029
Medication literacy	0.461 [0.410, 0.512]	0.026	0.259	17.527	<0.001**	1.290
Perceived stress	−1.337 [−1.505, −1.168]	0.086	−0.214	−15.580	<0.001**	1.158
Medication self-efficacy	−1.016 [−1.137, −0.896]	0.062	−0.238	−16.490	<0.001**	1.277
*R* ^2^	0.283					
Adjust *R*^2^	0.281					
*F*	192.594, *p* < 0.001					

Significant factors are in bold.

## Discussion

This study administered an electronic questionnaire survey to a sample of 4,288 children and adolescents across children’s hospitals in 18 cities throughout mainland China, constituting the largest dataset to date on medication behavior with substantial demographic representativeness. This study enhances the current literature on medication behavior among children and adolescents by elucidating the intricate factors influencing such behavior through the application of the TST. The results suggested that several factors were significantly correlated with reduced medication behavior scores, including younger age, lower BMI, the presence of chronic illnesses, recent residence in rural areas within the past 3 months, permanent residency registration in rural locations, absence of a separate living space, lower medication literacy scores, decreased perceived stress levels, and diminished medication self-efficacy levels. Among these factors, age, gender, medication literacy, perceived stress, and medication self-efficacy were identified as significant predictors of medication behavior.

Unhealthy housing conditions have been correlated with adverse health outcomes and are implicated in nearly 20% of deaths in Belgium (17). Our study similarly corroborated this association, revealing that children with access to private rooms exhibited significantly higher medication adherence. This finding underscores the critical role of housing quality in health outcomes. Nevertheless, housing improvements continue to be underprioritized in global public health agendas. Recently, the United Nations Special Rapporteur on the right to adequate housing underscored the urgency of the global housing affordability crisis, emphasizing the necessity for interventions to address its escalating trends (18). It is imperative for policymakers to acknowledge access to adequate housing as a crucial social determinant of health and to incorporate housing considerations as a fundamental component of public health strategies.

The TST framework addresses the fundamental challenge of balancing immediate benefits with long-term health outcomes, highlighting the enduring dilemma of modifying health behaviors in contrast to conventional health behavior theoretical models. In this study, we investigate medication literacy as a temporal contingency that encapsulates cognitive factors influencing intention. We evaluated the health literacy of Chinese children using the widely recognized health literacy assessment instrument in China, the HLS-SF12 (14). Our findings corroborate the existence of a correlation between medication literacy and medication behavior in children and adolescents, demonstrating that medication literacy reflects the recognition of the long-term benefits of proper medication behavior. This aligns with prior research linking medication literacy to improved medication adherence and overall quality of life (19–22). It is essential for healthcare providers to evaluate the accessibility and suitability of healthcare information and implement strategies aimed at enhancing medication literacy (23). Such efforts enable patients to better understand their conditions and treatment plans, thereby strengthening knowledge and confidence in health-related decision-making (24). The World Health Organization advocates for health literacy as a means to enhance population health, engage communities in health-related initiatives, and urge governments to fulfill their responsibilities in addressing health and health equity (25). The empirical findings from this study offer strategic guidance for the systematic integration of evidence-based health education into primary school curricula, with the goal of enhancing medication literacy. The insights derived from the data furnish a robust empirical foundation for the curricular integration of evidence-based pharmacological education within primary education systems. This integration aims to foster essential medication safety practices through effective pedagogical approaches.

Perceived stress constitutes a dynamic process that involves the appraisal of external stressors, typically measured using the PSS, a self-report instrument (26). The determinants of stress are multifaceted, with educational environments identified as a significant and extensively researched source, particularly among adolescent populations (27). Our study identifies an age-related escalation in perceived stress, potentially indicative of developmental advancements in self-awareness. Notably, an increased perception of stress is predictive of poorer medication adherence, reduced medication literacy, and decreased self-efficacy. Among adolescent populations, there is a strong correlation between stress perception and negative emotional states, which may impair task concentration and, consequently, adversely affect medication behaviors and subsequent health outcomes. To address the detrimental impact of perceived stress on medication behaviors, the implementation of a multi-tiered intervention framework is essential. It is imperative to evaluate adolescents’ stress levels and to strengthen their emotional regulation capacities within both educational and familial contexts. Furthermore, the deployment of smart devices, including wearable biosensors, offers a promising approach to monitoring medication adherence and perceived stress levels in everyday life.

The concept of self-efficacy, a well-established predictor of behavioral change and disease self-management, has been integrated into numerous social behavioral theories (28–30). It reflects an individual’s confidence in their capability to execute a specific behavior to attain the desired outcomes. In pharmacotherapy practice, medication self-efficacy specifically denotes one’s belief in successfully managing medication-related tasks. In our study, a higher level of self-efficacy was correlated with improved medication behaviors. This study indicates that children and adolescents possessing high medication self-efficacy are more predisposed to engage in and strategize their medication behavior. Our results are consistent with prior research demonstrating that self-efficacy is a predictor of medication adherence and the perceived burden of medication in illnesses management (31, 32). The predictive analysis of self-efficacy in relation to behavioral change has established a strong evidence-based foundation for intervention studies focused on self-efficacy. Healthcare professionals can employ strategies based on self-efficacy as a theoretical framework to enhance patients’ medication self-efficacy, thereby improving long-term medication adherence and mitigating the negative consequences associated with medication burden.

The findings serve as a foundation for the development and enhancement of health policies from a macroscopic perspective. By integrating behavioral factors into the design of health systems, these findings can significantly enhance the development of disease management policies and the implementation of tiered diagnosis and treatment strategies. The findings of this research provide substantial empirical evidence that can assist health authorities in refining guidelines for the long-term pharmacological management of pediatric patients. Additionally, these insights have the potential to support the integration of innovative tools into medical insurance reimbursement schemes or public health service initiatives, thereby optimizing the allocation of healthcare resources and enhancing the overall efficacy of health policy implementation.

## Limitation

This study possesses several limitations that must be considered when interpreting the results. Firstly, due to its cross-sectional survey design, it is incapable of establishing causal relationships between variables. This limitation affects the determination of the temporal sequence of outcomes and the directionality of causality. Nonetheless, it incorporates temporal contingency in accordance with TST theory, which accounts for the impact of long-term benefits. Secondly, there exists the possibility of hospital-based sampling bias, potentially limiting the capacity to understand self-management behaviors in contexts characterized by minimal supervision. The questionnaire requires a one-on-one collection method to guarantee timeliness and accuracy. This requirement contrasts with the one-to-many approach commonly utilized in schools or communities. To mitigate this, we adopted a nationwide stratified sampling approach across different tiers and regions. Thirdly, individuals who were unable to independently complete the questionnaire or comprehend its items were excluded from the participation. While this exclusion criterion is intended to enhance data accuracy, it may inadvertently introduce selection bias, given that some participants required assistance from their guardians or guidance from the investigator. Fourthly, most variables demonstrating statistically significant but less-than-small effect sizes may reflect the increased statistical power afforded by the large sample size rather than meaningful effects. These findings appear to lack practical or clinical significance, despite their statistical detection. Future research employing more precise measurement approaches or theoretically targeted interventions is warranted to establish whether robust effects exist. Fifthly, although the regression model achieved statistical significance, its explanatory power is relatively low. It suggests that clinically relevant predictors may have been omitted from the model. Additionally, the model’s limited explanatory power restricts its translational value for precision medicine applications. Before considering clinical implementation, it is imperative to conduct external validation in independent cohorts and to undertake efforts aimed at enhancing its explanatory power. Ultimately, the reliance on self-reported measures introduces potential biases, including reporting bias and social desirability bias. Notwithstanding these limitations, self-reported measures were selected due to their suitability for the online format of the study, as well as their flexibility and convenience. It is advisable for future research to explore alternative recruitment strategies to include a more diverse cohort of children, such as those from communities, schools, or through online platforms. This would provide a more comprehensive understanding of medication use behaviors across different contexts and health statuses. Additionally, we suggest that future research focus on validating the constructs used in our study specifically within pediatric populations to ensure the reliability and accuracy of the findings.

## Conclusion

The provision of safe medication for children constitutes a critical issue that has garnered substantial public attention. Presently, there exists a widespread societal consensus that the responsibility for managing children’s medication practices should predominantly rest with their guardians. For school-aged children and adolescents, who frequently do not benefit from continuous familial supervision, medication management is largely self-regulated. Considering the growing autonomy and heightened self-awareness of this demographic, it is crucial to augment their understanding and awareness of medication safety.

## Data Availability

The raw data is available for acquisition via email for academic purposes or other reasonable uses.
