# Supplementary material for: Current status and influencing factors of medication behavior among children and adolescents: a cross-sectional study in China
Source: Front Public Health. 2025 Jul 30;13:1583645. doi: 10.3389/fpubh.2025.1583645 (PMC12343645; doi:10.3389/fpubh.2025.1583645)
Supplement: Supplementary file 1 [file Data_Sheet_1.docx]

**Supplementary Material**

***Medication self-efficacy scales***

| **Items** | **Strongly Agree** | **Agree** | **Unsure** | **Disagree** | **Strong Disagree** |
| --- | --- | --- | --- | --- | --- |
| Taking medications on time every day is easy for me. | ○ | ○ | ○ | ○ | ○ |
| I can easily remember to take all my medicines in the correct way. | ○ | ○ | ○ | ○ | ○ |
| I can still take my medicine on time even if the administration changes. | ○ | ○ | ○ | ○ | ○ |
| I can still take my medications on time even when I am busy with schoolwork. | ○ | ○ | ○ | ○ | ○ |

***Medication behavior scales***

| **Items** | **Strongly Agree** | **Agree** | **Uncertain** | **Disagree** | **Strong Disagree** |
| --- | --- | --- | --- | --- | --- |
| **Label comprehension** | | | | | |
| I pay attention to the dosage and administration in the medicine instructions. | ○ | ○ | ○ | ○ | ○ |
| I pay attention to the contraindications in the medicine instructions. | ○ | ○ | ○ | ○ | ○ |
| I pay attention to the precautions in the medicine instructions. | ○ | ○ | ○ | ○ | ○ |
| I pay attention to the sections related to pediatric use in the medicine instructions. | ○ | ○ | ○ | ○ | ○ |
| **Recognition of adverse reactions** | | | | | |
| If central nervous system symptoms (e.g., headache, drowsiness) occurred after taking medication, I consider a potential link to the administered drug. | ○ | ○ | ○ | ○ | ○ |
| If gastrointestinal symptoms (e.g., nausea, diarrhea) occurred after taking medication, I consider a potential link to the administered drug. | ○ | ○ | ○ | ○ | ○ |
| If allergic reactions (e.g., rash, itching) occurred after taking medication, I consider a potential link to the administered drug. | ○ | ○ | ○ | ○ | ○ |
| When being prescribed medication, I inform the doctor of my history of drug allergies. | ○ | ○ | ○ | ○ | ○ |
| **Medication adherence** | | | | | |
| I often forget to take my medications. | ○ | ○ | ○ | ○ | ○ |
| If I feel that the medication is not effective after a few days, I will change the medication on my own. | ○ | ○ | ○ | ○ | ○ |
| I will adjust the dosage of the medication on my own. | ○ | ○ | ○ | ○ | ○ |
| **Administration methods** | | | | | |
| I have never paid attention to the interval between doses. | ○ | ○ | ○ | ○ | ○ |
| I always split my medications (capsules, tablets) by myself before taking them. | ○ | ○ | ○ | ○ | ○ |
| I take my medications according to the recommended method and temperature specified in the instructions. | ○ | ○ | ○ | ○ | ○ |
| I use liquids other than drinking water (e.g., tea, cola) to take my medications. | ○ | ○ | ○ | ○ | ○ |
| I pay attention to whether I should take my medications before, during, or after meals. | ○ | ○ | ○ | ○ | ○ |
| After missing a dose, I decide whether to take a make-up dose based on the doctor's instructions or the leaflet. | ○ | ○ | ○ | ○ | ○ |
| **Unsafe medication use** | | | | | |
| I take antibiotics (e.g., amoxicillin) without a doctor's prescription. | ○ | ○ | ○ | ○ | ○ |
| When going to the hospital for treatment, I proactively request that the doctor prescribe antibiotics. | ○ | ○ | ○ | ○ | ○ |
| I use antipyretic analgesics (e.g., ibuprofen) for extended periods (antipyretics for more than 3 days, analgesics for more than 5 days). | ○ | ○ | ○ | ○ | ○ |
| I take antipyretic analgesics (e.g., ibuprofen) more than 4 times in a single day. | ○ | ○ | ○ | ○ | ○ |
| I use antitussive agents without experiencing any symptoms. | ○ | ○ | ○ | ○ | ○ |
| I take melatonin. | ○ | ○ | ○ | ○ | ○ |
| I use medications long-term to improve memory. | ○ | ○ | ○ | ○ | ○ |
| **Difficulties in medication intake** |  |  |  |  |  |
| I feel anxious before taking medication. | ○ | ○ | ○ | ○ | ○ |
| I find it difficult to swallow medications (such as tablets or capsules). | ○ | ○ | ○ | ○ | ○ |
